# Imaging Plate Autoradiography for Ingested Anthropogenic Cesium-137 in Butterfly Bodies: Implications for the Biological Impacts of the Fukushima Nuclear Accident

**DOI:** 10.3390/life13051211

**Published:** 2023-05-18

**Authors:** Ko Sakauchi, Joji M. Otaki

**Affiliations:** The BCPH Unit of Molecular Physiology, Department of Chemistry, Biology and Marine Science, Faculty of Science, University of the Ryukyus, Nishihara 903-0213, Okinawa, Japan; yamatoshijimi@sm1044.skr.u-ryukyu.ac.jp

**Keywords:** cesium-137, exposure effect, field effect, Fukushima nuclear accident, imaging plate autoradiography, pale grass blue butterfly, radiation dose, transgenerational effect, *Zizeeria maha*

## Abstract

The Fukushima nuclear accident in March 2011 caused biological impacts on the pale grass blue butterfly *Zizeeria maha*. At least some of the impacts are likely mediated by the host plant, resulting in “field effects”. However, to obtain the whole picture of the impacts, direct exposure effects should also be evaluated. Here, we examined the distribution of experimentally ingested anthropogenic cesium-137 (^137^Cs) in adult butterfly bodies using imaging plate autoradiography. We showed that ^137^Cs ingested by larvae was incorporated into adult bodies and was biased to females, although the majority of ingested ^137^Cs was excreted in the pupal cuticle and excretory material during eclosion. ^137^Cs accumulation in adult bodies was the highest in the abdomen, followed by the thorax and other organs. These results suggest that ^137^Cs accumulation in reproductive organs may cause adverse transgenerational or maternal effects mediated by reactive oxygen species (ROS) on germ cells. ^137^Cs accumulation was detected in field individuals collected in September 2011 and September 2016 but not in May 2011, which is consistent with the abnormality dynamics known from previous studies. Taken together, these results contribute to an integrative understanding of the multifaceted biological effects of the Fukushima nuclear accident in the field.

## 1. Introduction

As of March 2023, 12 years have passed since the Fukushima nuclear accident occurred in March 2011. Large amounts of anthropogenic radioactive materials were released from the reactor into the surrounding environment [1,2,3,4,5,6]. These radionuclides were transported into the biosphere through ecological interactions among organisms. Most of the ground deposition of radionuclides remains in the ground, within 50 mm of the surface [7]. The major polluting radionuclide in the environment is now radioactive cesium-137 (^137^Cs) due to its relatively long half-life of 30 years, and it will take more than 300 years for environmental radiation doses to return to pre-accident levels [7]. Over a period of hundreds of years, ^137^Cs from the Fukushima nuclear accident will continue to circulate in the environment, passing generations of most organisms. Even before the accident, anthropogenic radionuclides from nuclear bomb tests and nuclear power plant accidents were detected in soil samples from various sites on the globe [8,9,10,11], but the Fukushima nuclear accident further added radionuclides to the environment [12].

The biological impacts of the Fukushima nuclear accident have been investigated in field-based surveys focusing on various organisms, including birds [13,14,15], insects [16,17,18,19,20], Japanese monkeys [21,22,23], intertidal species [24], and plants [25,26,27]. The organism that has been studied most extensively in this research field is probably the pale grass blue butterfly, *Zizeeria maha* (Lepidoptera: Lycaenidae) [28,29,30]. This butterfly is highly common in human habitats throughout Japan except Hokkaido [28,29]. It is monophagous, and its life history entirely depends on its host plant, the creeping wood sorrel *Oxalis corniculata* (Oxalidales: Oxalidaceae) [28,29]. This plant is very short in height, which necessitates the pale grass blue butterfly to live close to the ground surface. This butterfly species has the typical immature stages of holometabolous insects: egg, larva (first to fourth or fifth instars), prepupa, pupa, and adult [28,29,30]. To use as an experimental animal, a reliable rearing method has been established [31] with continuous improvements, under which approximately one month is required for one generation. Moreover, the pale grass blue butterfly had a previous case in which it was monitored in the field and was simultaneously subjected to experiments in the laboratory [32].

Taking advantage of these points, the pale grass blue butterfly has been investigated from multiple points of view in Fukushima research. Both field surveys and field-oriented laboratory experiments have been performed, including field sampling surveys [16,33,34,35,36,37,38], transgenerational rearing experiments [16,39,40,41,42], external irradiation experiments [16,42], and internal feeding experiments [16,39,40,41,42]. The collective evidence has demonstrated that butterfly populations in areas near Fukushima have been severely affected by radioactive pollution [43,44,45,46]. At the time of the accident, the pale grass blue butterfly was overwintering as larvae [38], and the initial exposure from not only ^137^Cs but also ^134^Cs and other short-half-life nuclides such as ^131^I immediately after the accident might have caused genetic or transgenerational damage, which could have manifested in morphological abnormalities in the next generation reared under nonradioactive conditions [16,17]. In addition, when larvae from Okinawa (the least contaminated area in Japan) were fed field-picked contaminated leaves from Fukushima (the most contaminated area in Japan), butterflies showed growth retardation, a decrease in the survival rate, and an increase in the morphological abnormality rate [16,39,40,41].

At first glance, the high sensitivity of the butterfly to relatively low-dose radiation exposure in Fukushima in the field reported above is not consistent with experimental exposure experiments on insects, which show high resistance against radiation exposure [17]. For example, to obtain morphological abnormalities in the wings of silkworm moths in 50% of treated individuals, 90 Gy was required [47]. Similarly, 50 Gy was required to obtain morphological and other abnormalities in a fruit fly [48]. The high resistance against direct radiation exposure in insects seems to be applicable to the pale grass blue butterfly based on Gurung et al. (2019) [49]. In that study, pure ^137^CsCl mixed with an artificial diet was given to larvae of this butterfly. Larvae of this butterfly do not show cannibalization, and they only consumed the ^137^Cs-containng diet. As a result, a larva consumed, in total, 149 MBq/kg larva (wet) at maximum throughout its life, and no statistically significant difference in rates of pupation, eclosion, and survival were observed between larval groups that consumed either radioactive or nonradioactive cesium chloride [49]. Although equivalence of the two groups was not tested, this test was performed at three different concentration levels, resulting in no significant difference [49]. Moreover, no difference was detected in rates of pupation, eclosion, and survival and in forewing size among different levels of concentrations over seven orders of magnitude [49]. These results were in contrast to previous field-based studies. However, these results are consistent with a similar study in Tanaka et al. (2020) [50], in which ^137^CsCl was given to silkworms. These key experiments [49,50] have demonstrated that these lepidopteran insects and possibly other insects are resistant against relatively low-dose radioactive exposure itself under experimental conditions in the laboratory despite their vulnerability in the field. This discrepancy between field-based and laboratory-based results is called the field–laboratory paradox [51], which led us to propose that the biological effects on the pale grass blue butterfly in Fukushima in the field are mediated by “field effects”, one of which is mediated by ecological interactions with its host plant [43,44,45,46,51]. This field effect hypothesis has been supported by a series of nutritional [52], metabolomic [53,54], and toxicological analyses [55]. Other types of field effects, such as synergistic effects, may also be important [51]. Nevertheless, the direct exposure effects of radioactive materials should also be studied further.

In a previous ^137^Cs-feeding study [49], the radioactivity concentrations in prepupae were measured to confirm that ^137^Cs was ingested and retained in their bodies. Regrettably, adult butterflies were not subjected to measurements of radioactivity concentrations. As a result, we do not know whether ^137^Cs in prepupae was kept in the digestive tracts without absorption into the body or whether ^137^Cs was entirely absorbed into the body. In the latter case, ^137^Cs may be retained in adult bodies and accumulate in certain parts of the body, which could make butterflies more vulnerable to radioactive exposure. It is important to determine these points, considering that ^137^Cs emits γ-rays and β-rays and then becomes stable as ^137^Ba and that β-rays are likely absorbed regionally, although the small body size may make the distribution of tissue/organ accumulation of radioactivity less important than larger organisms such as vertebrates.

Meanwhile, numerous dosimetric studies on wild organisms in Fukushima have been reported. In vertebrates, a common tendency of muscle to accumulate ^137^Cs has been reported in many studies. Examples are cattle [56,57], wild boars [58,59,60,61], Japanese monkeys [62,63], and fishes [64,65]. Accumulation of ^137^Cs has been reported in various field-collected insects, spiders, and other invertebrates [66,67,68,69,70,71,72,73,74,75,76,77]. In the case of earthworms, 95% of ^137^Cs was found in the intestine, and only 2.6% was found in body wall muscles [78]. These results can be informative, but to the best of our knowledge, the distribution patterns of radioactive materials in insect bodies in Fukushima have not yet been examined. One reason for this scarcity of studies on the distribution of radionuclides in insect bodies may be due to their relatively small body size. Body parts of insects are even smaller, and it is often difficult to obtain sufficient sample volume to subject to a germanium semiconductor detector. The pale grass blue butterfly is not an exception. It has not yet been fully studied dosimetrically, not to mention the distribution patterns in the bodies.

In the present study, we aimed to clarify the accumulation and distribution of ^137^Cs in butterfly bodies using imaging plate (IP) autoradiography, which is suitable for small samples such as the pale grass blue butterfly and its body parts. Imaging plate autoradiography is a powerful technique to visually identify radioactive materials in the body of organisms and has been performed for field samples, including earthworms [78], contaminated soil particles [79], plants [72,80,81,82,83,84], pig skeletal muscle [85], aquatic insects [68], and spiders [77]. In the present study, samples were obtained from our previous studies, a ^137^Cs-feeding experiment [49], and field sampling surveys in 2011 [16,17] and 2016 [34]. The former samples were internally exposed by ingestion of ^137^Cs added to an artificial diet, and the latter samples were externally and internally exposed in the field. The present study is likely the first case of using imaging plate autoradiography for both experimentally ^137^Cs-fed and field-collected insects in Fukushima. Based on the present results, we discuss the potential biological effects of accumulated ^137^Cs on the pale grass blue butterfly in the field.

## 2. Materials and Methods

### 2.1. Butterflies Reared with a ^137^Cs-Containing Artificial Diet

Butterfly samples that were reared in the laboratory of the RI (radioisotope) Facility at the University of the Ryukyus were obtained from a previous study, in which late third-instar larvae were fed an artificial diet containing ^137^CsCl [49]. This feeding experiment was performed in 2016. The levels of ^137^CsCl concentrations varied by several orders of magnitude and were named H0 (the ingested ^137^Cs radioactivity was 0 Bq/larva, equivalent to 0 Bq/kg larva), H3 (4.94 Bq/larva, 1.49 × 10^5^ Bq/kg larva), H4 (49.4 Bq/larva, 1.49 × 10^6^ Bq/kg larva), H5 (494 Bq/larva, 1.49 × 10^7^ Bq/kg larva), and H6 (4940 Bq/larva, 1.49 × 10^8^ Bq/kg larva) [49]. H3 is the level of field contamination at the time of the accident in Fukushima, and H6 is the maximum concentration used in the previous study [49].

Adult male (*n* = 15) and female (*n* = 15) individuals from the previous study (dead dried specimens) were obtained; three individuals from each CsCl concentration level (H0–H6) in each sex. This small sample size was unavoidable because most specimens did not have intact body parts due to uncontrolled preservation of the specimens and because individuals with possible morphological abnormalities and those with very small size were excluded. To examine the body parts, after imaging plate exposure using the whole body, H6 male and female individuals were subjected to dissection of body parts for subsequent imaging plate analysis. To do so, the whole-body dried specimens were first confined in a small container with wet paper tissue for 1 d before dissection. Then, the following body parts were separated: four wings, two antennae, six legs, head, thorax, and abdomen. They were redried with desiccants for more than one week. Pupal cuticle cases (exuviae) shed after eclosion from males (*n* = 8) and females (*n* = 15) were also examined. Dried pupae (died likely immediately before eclosion) were also examined (*n* = 15 in total; *n* = 3 from each ingestion level), although they were “adults” inside the pupal cuticle case after apolysis.

### 2.2. Butterflies Collected from Contaminated Fields

Butterfly samples collected from contaminated fields were obtained from previous studies [16,17,33]. We used 82 butterflies (dead dried specimens), including males (*n* = 42) and females (*n* = 40), collected in May 2011, September 2011, and September 2016 from nine cities in Fukushima and Ibaraki prefectures, including the “standard” seven collection localities (Fukushima, Motomiya, Hirono, Iwaki, Takahagi, Mito, and Tsukuba). We used samples whose whole bodies were preserved well. We did not find any significant correlation between ground radiation dose rates and photostimulated luminescence (PSL) values (*p* > 0.05) in this study. This is probably because ground depositions of radionuclides are not uniform even within a small area. Therefore, we did not pay attention to the pollution levels of the collection locality in this study.

From the May 2011 field collection [16,17], we used two females from Fukushima (Fukushima Prefecture), one female from Motomiya (Fukushima Prefecture), four males from Hirono (Fukushima Prefecture), one male and three females from Iwaki (Fukushima Prefecture), one male from Takahagi (Ibaraki Prefecture), and one female from Mito (Ibaraki Prefecture). From the September 2011 field collection [16,17], we used four males from Fukushima, six females from Motomiya, one male and two females from Hirono, and three males and three females from Iwaki. From the September 2016 field collection [33], we used four males and one female from Fukushima, three males and one female from Iitate (Fukushima Prefecture), six males and three females from Katsurao (Fukushima Prefecture), three males and four females from Tomioka (Fukushima Prefecture), six males and six females from Hirono, and six males and seven females from Iwaki. As a control group, we used six males and eight females collected in Kobe in October 2011 (Hyogo Prefecture).

### 2.3. Imaging Plate Exposure

As output values of imaging plate autoradiography, PSL values were obtained throughout this study. To do so, we employed a Cytiva BAS SR 2025 E imaging plate (Tokyo, Japan). A whole plate was cut into two plates (100 × 80 mm and 100 × 88 mm) for convenience, and both plates were used indiscriminately. Before sample exposure, the plates were subjected to a Fuji Photo Film eraser FUJIX BAS 1000 (Tokyo, Japan), and we confirmed that the plate signals were completely erased (average intensity ≤ 0.04 with 950 V and 100 μm).

**Figure 1:**
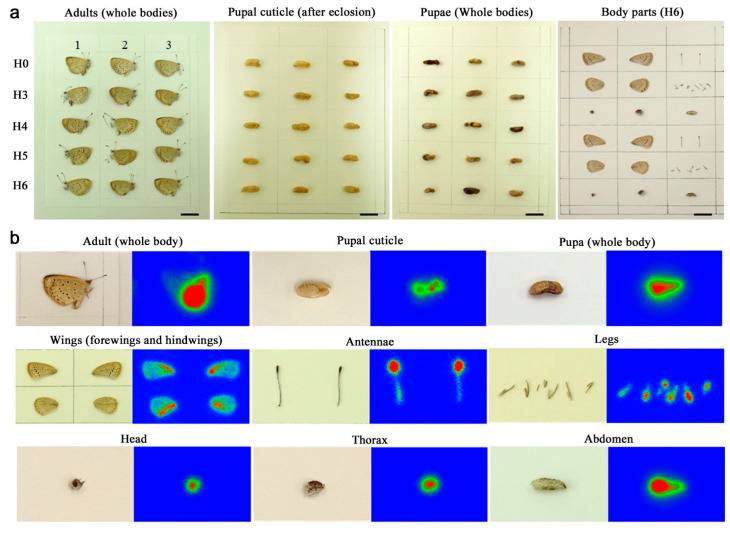
Imaging plate autoradiography of the pale grass blue butterfly *Zizeeria maha*. Larvae were fed a ^137^Cs-containing artificial diet, and whole-body adults, pupal cuticle cases, whole-body pupae, and adult body parts were subjected to this analysis. (**a**) Examples of layout on an imaging plate. In the three panels of adults, pupal cuticle, and pupae, samples were aligned according to the ^137^Cs radioactivity groups (the ingestion levels at the larval stage), H0–H6. All samples except pupae shown here are females. Pupal sex is unknown. Scale bars, 10 mm. (**b**) Examples of the imaging plate images of H6 samples of a whole-body adult, a pupal cuticle, a pupa, and adult body parts. In these examples, the pupal cuticle and pupa were exposed for 2 d (two days), and others were exposed for 6 d. Image contrast was adjusted to clarify the ^137^Cs gradient. Red areas indicate the highest PSL value.

Samples were aligned on thin paper according to the ingestion levels (H0, H3, H4, H5, and H6) (Figure 1a). They were moved onto the plate together with the paper. For the whole-body exposures, directions of adult individuals on the plate were determined to maximize contact surface of samples with the imaging plate (either right or left direction). The directions of the pupal case were identical to those of the corresponding adults. All pupae and body parts were set in the same directions. Especially for the whole-body exposures, dried specimens had thicknesses of a few millimeters, but we did not press them because they were so fragile that they would be destroyed when pressed. When body parts were accidentally removed during this operation, these parts were also made to contact the plate as much as possible. A portion of the imaging plate was always reserved without samples to obtain background data.

The plate with samples was transferred into a shielding apparatus, which was made from lead blocks (100 × 200 × 50 mm). Inside the block shield, an acryl case (10 mm thickness; 190 × 110 × 30 mm inside) was set to shield β-rays from the lead blocks. A Shinwa Rules thermometer (Sanjo, Niigata, Japan) was set inside the shielding apparatus to monitor temperatures. Imaging plate exposure in the shielding apparatus was conducted when temperatures were 17–24 °C (November 2021–February 2022) for ^137^Cs-fed individuals and 24–29 °C (May–October 2022) for field-caught individuals to avoid latent image fading at higher temperatures.

### 2.4. Exposure Periods

The 8-d (eight-day) whole-body exposure against imaging plates from H6 females, which had the largest radioactivity concentration, hit the maximum detection limit (100,000 PSL). The longest exposure days were thus set at 8 d. For the whole body, imaging plate exposure was made in the periods of 1, 2, 4, 6, and 8 d, and 2-d exposure results were mainly used for analyses. For the pupal cuticle cases, pupae, body parts, and field-caught whole adult bodies, imaging plate exposures were made for 2 d. In addition, 6-d exposures were also made to obtain clearer images of the whole adult bodies and body parts (Figure 1b). For the ^137^Cs-fed and field-caught individuals, exposure was performed once and five times per individual, respectively.

### 2.5. Data Acquisition and Analyses

Postexposure operations were executed under dim light to minimize fading. The exposed imaging plate was scanned within 1.5 min after removal of samples using a Cytiva scanner Typhoon FLA 9500 with a maximum sensitivity of 950 V and a minimum pixel size of 25 μm. To do so, the Cytiva reading software Typhoon FLA 9000 and the Cytiva analysis software ImageQuant TL Analysis Toolbox were used. Examples of autoradiographic images are shown in Figure 1b. Quantitative intensity data were obtained as PSL values (Supplementary Tables S1 and S2). The present study uses PSL values, but their relationship to becquerel is discussed in Appendix A. To obtain the PSL values of the object of interest, contrast values and analysis areas were visually determined, and background values were subtracted because any “signal” below background values should be considered noise, as recommended by the manufacturer. Detection limit (i.e., discrimination limit of “signal” from background “noise”) associated with the method performed in this study is discussed in Appendix B. When the latent image was not seen, a region of interest was determined in reference to the sample picture before exposure. The H0 samples (three males and three females), which had no ^137^Cs feeding, were considered as a control group. The relationship between the whole body and body parts is discussed in Appendix C.

The field-caught individuals showed fluctuations in PSL values due to the low-level accumulation of radionuclides and the detection limit of our system. To cope with this situation, an individual was measured five times. Negative values (two to five results) were discarded from the five analytical results per individual, which is reasonable because negative values were likely obtained due to fluctuations of the detection system. Positive values may also be obtained due to fluctuations but should be more frequent than negative values when the signals are sufficiently larger than background noise, due to ^137^Cs of a sample. In that case, distribution of measurement values from a single individuals could be assumed to be normal or nearly normal. Positive results in each individual were thus subjected to one-sided Welch’s *t*-tests against the results of the control groups (collected in Kobe, October 2011). That is, each individual sample was evaluated to determine whether its PSL values were significantly larger than those of the control groups (“positive” individuals) or not (“negative” individuals). The number of positive and negative individuals was further evaluated in accordance with Fisher’s exact test. The feasibility of using these positive values is discussed in Appendix D. For the control group (Kobe samples), eight males and eight females were measured five times per individual, but two males were excluded because they showed smaller values than background values in all measurements. Because ^40^K levels in butterflies should be nearly constant, the higher PSL values in the field-caught samples were considered the result of ^137^Cs accumulation in the body. The control samples could contain ^137^Cs, albeit at very low levels, but such levels were considered “background” in the present study.

For weight measurements, we used a Mettler-Toledo MX5 Microbalance (Columbus, OH, USA) located outside the RI Facility of the University of the Ryukyus. Because the ^137^Cs-fed individuals were preserved in the RI Facility and they were not allowed to be taken out from the RI Facility, the H1–H6 individuals were not measured, but H0 individuals were measured and used as reference weight values (Supplementary Table S3).

### 2.6. Statistical Analyses

We used R version 4.1.2 (R Foundation for Statistical Computing, Vienna, Austria, 2021), JSTAT 16.1 (Yokohama, Japan, 2014), and Microsoft Excel (Office 365) for statistical analyses. In accordance with the Shapiro–Wilk test for normality, Spearman correlation analysis was performed to obtain the Spearman rank correlation coefficient *ρ* and its associated *p*-value. For comparison of two groups, either Welch’s *t*-test or Student’s *t*-test (bi-sided test unless otherwise indicated) was performed depending on potential unequal or equal variance. The field-caught samples were categorized into either a group of individuals with significantly higher PSL values than the control group (“positive” group) or a group of individuals without significantly higher PSL values (“negative” group) using one-sided Welch’s *t*-test. With these two categorical data, Fisher’s exact test was performed. Throughout this study, we considered *p* < 0.05 statistically significant. For simplicity, *p*-values were not adjusted by Bonferroni or other methods. Microsoft Excel (Office 365) was used to obtain linear equations with *R*^2^ values and to make graphs for data visualization.

## 3. Results

### 3.1. Adult Whole-Body Samples

**Figure 2:**
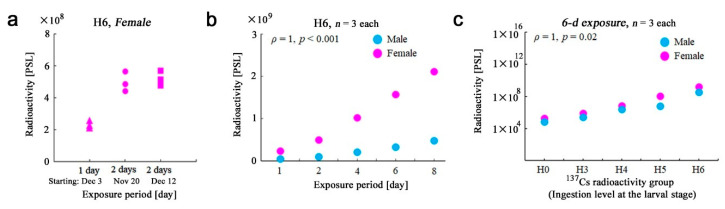
Radioactivity levels of adult whole-body samples (*n* = 3 at each exposure). (**a**) Radioactivity after 1-d or 2-d exposure of H6 female samples. (**b**) Radioactivity of 1-d to 8-d exposures of H6 male and female samples. The mean values of three measurements at each exposure time are plotted. Both male and female samples show a Spearman correlation coefficient *ρ* = 1 (*p* < 0.001). (**c**) Radioactivity of exposure of H0–H6 samples. Mean values of three measurements at each ^137^Cs radioactivity group (ingestion level at the larval stage) are plotted. Note that both axes are logarithmic scales. Both male and female samples show a Spearman correlation coefficient *ρ* = 1 (*p* = 0.02).

As a pilot experiment, we first tested whether H6 female individuals (the highest level of ^137^Cs ingestion) exhibited detectable radioactivity on the imaging plate after 1-d exposure (Supplementary Table S1). We readily detected radioactivity from these female butterflies (*n* = 3) (Figure 2a). This result demonstrated that at least these three butterflies accumulated ^137^Cs in their bodies and ensured the feasibility of our method of imaging plate autoradiography applied to the butterfly system. As expected, 2-d exposure of the same set of samples doubled the PSL signals (*n* = 3) (Figure 2a). Furthermore, the same sample set was exposed for 2 d 22 days later, resulting in virtually the same PSL values without the detection of radioactive decay (*n* = 3) (Figure 2a). These results justified the use of imaging plates for 2-d exposures and the treatment of radioactive decay of ^137^Cs as negligible in this experimental system for a period of a few months.

To examine the linearity of radioactivity signal detection, using H6 males (*n* = 3) and females (*n* = 3), we changed the exposure periods from 1 d to 8 d (Supplementary Table S1). As expected, we obtained a linear increase in PSL values in both sexes (*ρ* = 1, *p* < 0.001) (Figure 2b), demonstrating the high accuracy of this method. Somewhat unexpectedly, sex differences were notable; females had radioactivity much higher than males. This could be because females are larger in size than males in this species [28,29,30,31]. We then tested if different levels of ^137^Cs ingestion (H0–H6) were carried onto the adult bodies and if their different levels could be accurately detected by this method using the 6-d exposure. Linear dose responses in both sexes in response to an increase in ^137^Cs in an artificial diet in a double logarithmic plot were detected over the range of 10^5^ in both axes (*ρ* = 1, *p* = 0.02) (Figure 2c), showing that ^137^Cs from larval feeding was assimilated in the adult bodies dose-dependently in a scale-free manner over the range examined here. In other words, ^137^Cs ingested in larvae was carried onto adulthood without any maximum limit of ^137^Cs absorption into the body over this range. Again, sex differences were notable at all levels of ingestion.

### 3.2. Sex Differences in the Whole-Body Samples

**Figure 3:**
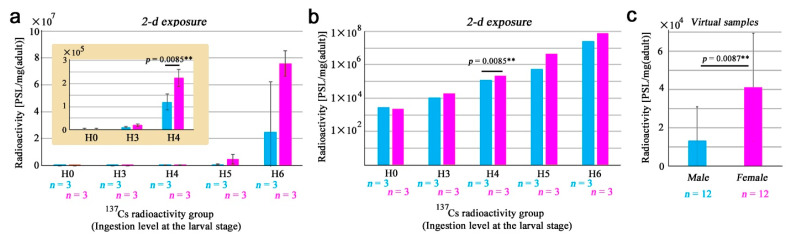
Weight-adjusted radioactivity levels in adult whole-body samples. Blue and pink bars indicate males and females, respectively. Asterisks indicate statistical significance: **, *p* < 0.01 (Welch’s *t*-test). Error bars indicate standard deviation. (**a**) Single logarithmic expression. Note that the *x*-axis is logarithmic due to tenfold changes in ingestion levels at the larval stage. Inset shows H0–H4 at different scales of the *y*-axis. (**b**) Double logarithmic expression. Data are identical to (**a**). Only H4 was significantly different between sexes. (**c**) Virtual samples using H3–H6 samples (excluding H0) adjusted to the H3 levels (*n* = 12 for each sex).

Based on the results above and just for convenience, we mainly employed the 2-d exposure period in subsequent analyses (Supplementary Table S1). Here, sex differences were statistically examined using adult butterfly samples from the entire range of ingestion levels, H0–H6. To do so, PSL values were divided by body weights for standardization. Both males and females showed exponential responses in a single logarithmic graph (Figure 3a). Both males and females responded linearly to an increase in ^137^Cs in the double logarithmic graph (Figure 3b); we obtained *y* = 0.96*x* + 2.24 (*R*^2^ = 0.97) for males and *y* = 1.15*x* + 2.07 (*R*^2^ = 1.00) for females, where *x* and *y* are expressed in the common logarithm (log_10_) of the original variables. Despite the weight standardization, at all ingestion levels (except H0), females had larger values than males. However, partly due to a small number of samples with large SD (standard deviation) bars, sex differences were not significant except at H4 (*p* = 0.0085; Welch’s *t*-test). Nonetheless, the female-biased incorporation of ^137^Cs at other ingestion levels (H3, H5, and H6) appeared to be a reality beyond the sexual weight difference.

To further explore this possibility, we “adjusted” the original data at different ingestion levels (H3, H4, H5, and H6) of ^137^Cs into a single level, H3, simply by changing orders of magnitude. This is operationally possible and reasonable because of a “linear” increase (*R*^2^ = 0.97 for males and *R*^2^ = 1.00 for females) in PSL signals in response to the radioactivity concentration of ^137^Cs in the artificial diet (i.e., an exponential response to an exponential increase). Because the coefficients of the linear equations were nearly one (0.96 for males and 1.15 for females), we used a conversion factor of 1.00 for this adjustment. This operation made it possible to compare sexes statistically with a reasonable number of virtual samples (*n* = 12 for each sex). Using these virtual samples, we obtained 13,107.35 ± 17,880.88 (mean ± SD) PSL/mg for males and 41,009.89 ± 28,418.60 PSL/mg for females (Figure 3c). These values showed a statistically significant difference (*p* = 0.0087; Student’s *t*-test) (Figure 3c). These results suggest that the female-biased PSL values are not simply due to female-biased weight and seem to originate from sex-specific morphological and physiological traits.

### 3.3. Pupal Cuticles and Their Sex Differences

**Figure 4:**
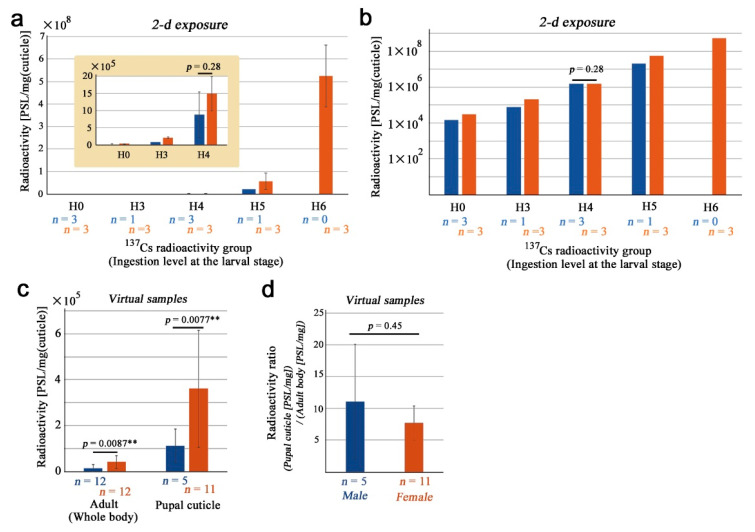
Weight-adjusted radioactivity levels in pupal cuticle (exuviae) samples. Dark blue and orange bars indicate males and females, respectively. (**a**) Single logarithmic expression. Note that the *x*-axis is logarithmic due to tenfold changes in ingestion levels at the larval stage. Inset shows H0–H4 at different scales of the *y*-axis. Error bars indicate standard deviation. (**b**) Double logarithmic expression. Data are identical to (**a**). H4 (having a minimum number of samples required for *t*-test) was not significantly different between sexes. (**c**) Virtual samples using H3–H6 samples (excluding H0) adjusted to the H3 levels (*n* = 5 for males and *n* = 11 for females; one female H5 sample was discarded as an outlier). For comparison, adult virtual samples are also shown. Asterisks indicate statistical significance: **, *p* < 0.01 (Welch’s *t*-test). (**d**) Radioactivity ratio (pupal cuticle divided by adult whole body) using virtual samples.

Here, we examined pupal cuticle cases (Supplementary Table S1). Pupal cuticle samples were similar to the adult samples in terms of PSL distributions over the ingestion levels (Figure 4a,b). In a single logarithmic graph, the responses were exponential (Figure 4a). Although there seemed to be a sexual difference at all ingestion levels, the H4 samples here did not show a significant difference (*p* = 0.28; Welch’s *t*-test). In a double logarithmic graph, a linear increase in PSL values was observed in response to an increase in ^137^Cs concentrations ingested at the larval stage even after standardization by weights of pupal cuticle cases (Figure 4b). In a double logarithmic plot, we obtained *y* = 1.08*x* + 2.94 (*R*^2^ = 0.99) for males and *y* = 1.09*x* + 3.21 (*R*^2^ = 0.99) for females.

Because of the linearity of PSL data in response to the ingested amount of ^137^Cs at the larval stage, we adjusted the original data by changing orders of magnitude, as in the adult whole bodies, to the H3 level to increase the number of virtual samples. This operation was justified by the high *R*^2^ values and by the fact that coefficients were nearly 1.00. For these virtual samples, we obtained 110,155.55 ± 73,106.41 PSL/mg for males (*n* = 5) and 360,232.05 ± 255,618.13 PSL/mg for females (*n* = 11) (Figure 4c). Their difference was statistically significant (*p* = 0.0077; Welch’s *t*-test) (Figure 4c), demonstrating that females inherently accumulated ^137^Cs more than males not only in the adult bodies but also in the pupal cuticle cases. When the radioactivity of the pupal cuticle (PSL/mg) was divided by that of the adult body (PSL/mg), we obtained ratios of 11.06 ± 9.04 for males and 7.67 ± 2.72 for females (Figure 4d). These ratios indicated that the pupal cuticle accumulated ^137^Cs much more than the body proper. The sex difference was not significant in these ratios (*p* = 0.45; Welch’s *t*-test) (Figure 4d).

### 3.4. Pupae

**Figure 5:**
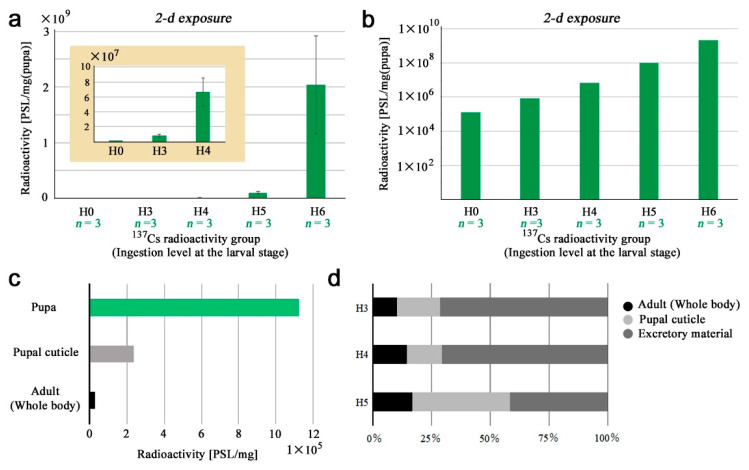
Weight-adjusted radioactivity levels in pupal whole-body samples. (**a**) Single logarithmic expression. Note that the *x*-axis is logarithmic due to tenfold changes in ingestion levels at the larval stage. Inset shows H0–H4 at different scales of the *y*-axis. Error bars indicate standard deviation. (**b**) Double logarithmic expression. Data are identical to (**a**). (**c**) Comparison among the adult whole body, pupal cuticle, and pupa. Mean values of male and female mean values of adjusted data at the H3 level were used for this comparison. (**d**) ^137^Cs distribution in a pupa at different radiation levels (H3–H5). Mean values of male and female mean values were used for this graph. H6 was excluded because of a lack of male pupal cuticle samples.

Here, we examined whole pupae (Supplementary Table S1). Pupae before adult wing development cannot be sexed easily, and thus, we did not differentiate sexes here. The accumulation of ^137^Cs was exponential in a single logarithmic graph (Figure 5a) and linear in the double logarithmic graph (Figure 5b) in response to the exponential ingestion levels of ^137^Cs. In the latter, we obtained *y* = 1.05*x* + 3.87 (*R*^2^ = 0.99). When data from all ingestion levels (except H0) were adjusted to the H3 level, as in the previous sections, we obtained 1,123,282.99 ± 694,945.55 PSL/mg (*n* = 12) without sexual identification (Figure 5c). This value was much larger than those of the adult whole body and the pupal case (both sexes combined) (Figure 5c). The mean ratio of adults to pupae (i.e., adult retention rate) was calculated to be 0.1405 ± 0.0294 (*n* = 4; the number of ingestion levels, H3–H6) (Appendix E), indicating that the majority of the pupal ^137^Cs (85.95%) was excreted upon eclosion to the adult stage.

Because a whole pupa should be a summation of the adult whole body, pupal cuticle, and excretory material (excreted during eclosion), we obtained how ^137^Cs was distributed in these parts at each ingestion level (H3–H5). It appeared that most ^137^Cs was excreted upon eclosion at H3 and H4 (Figure 5d). At H5, however, excretory material may be saturated with ^137^Cs, and the accumulation in the pupal cuticle was notable.

### 3.5. Body Parts of Adults

**Figure 6:**
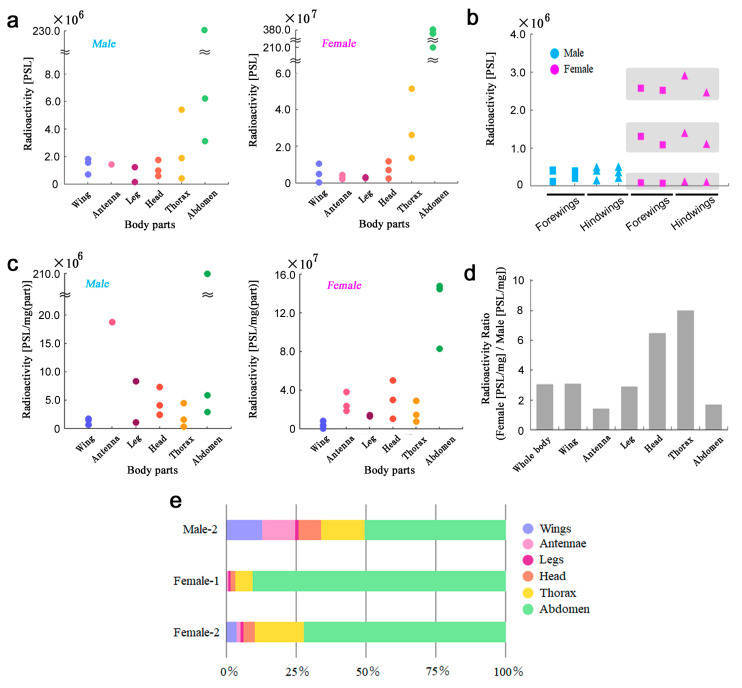
Radioactivity levels in six body parts of the H6 samples. (**a**) PSL values. Males (**left**) and females (**right**). (**b**) Wings. Four dots (two rectangular and two triangular dots) are shaded together in females, indicating that they are from the same individual. (**c**) Weight-adjusted PSL values. Males (**left**) and females (**right**). (**d**) Ratios of the weight-adjusted female PSL values to the weight-adjusted male PSL values. (**e**) Relative levels among the six body parts examined in the present study. These three samples had all body parts.

Here, we examined six body parts of the H6 samples (Supplementary Table S2). The abdomen and the thorax accumulated more than other body parts tested in males (*n* = 3) (Figure 6a, left) and females (*n* = 3) (Figure 6a, right). Four wings (two forewings and two hindwings) equally accumulated ^137^Cs in males (*n* = 3) and females (*n* = 3) (Figure 6b). When standardized by weight, radiation was still high in the abdomen but not the thorax (Figure 6c).

To understand sexual differences in accumulation in body parts, we calculated the female/male ratios in body parts. Interestingly, the female/male ratios were more than one in all body parts, indicating that females accumulated more than males in all the body parts examined here (Figure 6d). In these ratios, the thorax and the head were notable, but the abdomen was not very much in comparison to other body parts, suggesting that the abdomen in both sexes was the site of high accumulation (Figure 6d). This body part distribution pattern varied to some extent among individuals and sexes. In a female individual, approximately 90% of radioactivity was detected in the abdomen (Female-1 in Figure 6e). In a different female individual, the bias to the abdomen was not as high (Female-2 in Figure 6e). In a male individual, the bias to the abdomen was further reduced to approximately 50% (Male-2 in Figure 6e).

### 3.6. Field-Caught Adults

**Figure 7:**
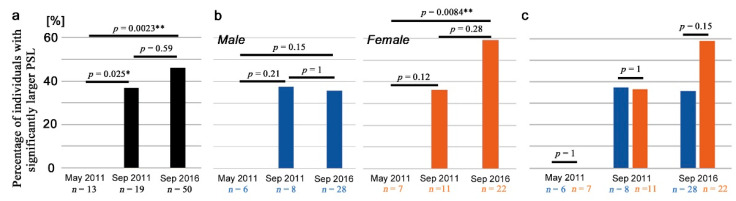
Comparison of field samples. The percentages of positive individuals with significantly larger PSL values than those of the control group are shown. Dark blue and orange bars indicate males and females, respectively. Asterisks indicate statistical significance: *, *p* < 0.05; **, *p* < 0.01 (Fisher’s exact test). (**a**) Comparisons between sampling dates using all samples (males and females together). (**b**) Comparisons between sampling dates using male (**left**) or female (**right**) samples. (**c**) Comparisons between male and female samples on the same sampling dates.

Here, we examined field-collected samples from contaminated areas. Each individual sample was evaluated to determine whether its PSL values were significantly larger than those of the control groups (“positive” individuals) or not (“negative” individuals). Without sex differentiation, the percentage of positive individuals significantly increased from May 2011 to September 2011 (*p* = 0.025; Fisher’s exact test here and hereafter in this section) and from May 2011 to September 2016 (*p* = 0.0023) (Figure 7a; Table 1). The difference from September 2011 to September 2016 was not statistically significant (*p* = 0.59) (Figure 7a). Similar comparisons using only male individuals did not show any significant difference (Figure 7b left), but using only female individuals, a statistically significant difference was obtained between May 2011 and September 2016 (*p* = 0.0084) (Figure 7b right). Direct comparisons between male and female samples on the same sampling date did not show any statistically significant difference, although there was a female-biased trend in samples collected in September 2016 (Figure 7c).

## 4. Discussion

### 4.1. Significance of This Study

This study is the first experimental demonstration of the accumulation of ingested ^137^Cs in butterfly bodies. Although accumulation of ^137^Cs was detected in prepupae using a germanium semiconductor detector in a previous study in which ^137^CsCl was administered to butterfly larvae via an artificial diet [49], there were three problems associated with the use of prepupae. First, because prepupal radioactivity may not necessarily be carried onto the adult body, it was difficult to estimate radioactivity concentrations of adults from those of prepupae. Second, to use a germanium detector, 10–19 individuals for each concentration level were homogenized and used for measurements to make sufficient sample volume due to the small volume of this butterfly [49]. As a result, only averaged values of multiple individuals were obtained. Third, it is very difficult, if not impossible, to identify sexes at the pupal and prepupal stages based on morphological traits in this butterfly. The homogenized samples unavoidably contained both sexes, making it impossible to detect sexually differentiated accumulation, if any.

The present study solved these problems using imaging plate autoradiography. We successfully detected radioactivity from individual adult butterflies, demonstrating that ^137^Cs ingested at the larval stage was carried onto adult bodies. At the pupal stage, the pupal cuticle appeared to accumulate ^137^Cs, and the majority of ^137^Cs (85.95%) was excreted at the time of eclosion, but some portions (14.05%) were retained in adult bodies. Ingested ^137^Cs accumulated in the body without saturation, at least in our experimental range, up to H6. We also showed that accumulation was more prominent in females. Furthermore, the critical limitation of using a germanium detector for small organisms was overcome in the present study by performing imaging plate autoradiography not only for individual adult butterflies but also for adult body parts, some of which are on the order of millimeters.

Importantly, this study is also the first demonstration that butterflies collected from the field accumulated anthropogenic ^137^Cs from the Fukushima nuclear accident in the body. Field-collected butterfly samples were important specimens that should not be homogenized for a germanium detector. In the present study, we used imaging plate autoradiography, and thus, individual information was obtained without destroying invaluable specimens. It can be safely assumed that ^40^K was equally contained among field-caught adult individuals and that there is no other radionuclide except ^137^Cs that could potentially be detected by the present analysis. The finding that field butterflies are directly contaminated with ^137^Cs, together with experimental ^137^Cs-feeding data, makes it possible to differentiate the potential biological impacts of direct internal exposure mainly mediated by reactive oxygen species (ROS) from the field effects partly mediated by toxic secondary metabolites in the host plant. That is, it has been shown by a series of nutritional, metabolomic, and toxicological studies that the host plant *O. corniculata* responds to low-dose radiation by changing levels of nutrients and secondary metabolites including toxicants to larvae [51,52,53,54], resulting in a large impact on butterflies. For example, lauric acid is upregulated in *O. corniculata* upon irradiation [53] and is toxic to larvae when ingested at high levels [55]. Antioxidants are also upregulated [53]. This field effect is likely an important part of the biological impacts of the Fukushima nuclear accident in addition to the direct radiation exposure that generates ROS.

In general, at the time of eclosion, the adult digestive tract is free from ingested materials. Therefore, the radioactivity levels detected in the fresh adults should come from the assimilated ^137^Cs in the body from larval ingestion. The radioactivity levels detected in the field-caught adults may also come from flower nectar and water that may be ingested at the adult stage. External adsorption of radionuclides on the surface of butterfly bodies is probably rare, considering that adult butterflies simply fly or rest repeatedly. Because the field-caught adults were at the level of detection limits of the imaging plate analysis, distributions in body parts could not be resolved in the field-caught samples. However, it is reasonable to speculate that ^137^Cs in the field samples is distributed in body parts similarly to the experimental ^137^Cs-fed butterflies.

Just to be sure, the present method has disadvantages over other methods: imaging plate autoradiography cannot directly evaluate radioactivity concentrations (Bq/kg), cannot differentiate radionuclide species, cannot efficiently detect radiation from three-dimensional objects, and cannot evaluate absorbed doses. More technically, imaging plate autoradiography is said to be unfavorable for quantitative analysis due to the fading of latent images over time after exposure. Complementary use of other methods is necessary to obtain a whole picture of radiation exposure and its effects in organisms.

### 4.2. Female Bias and Accumulation in Body Parts

We showed that females accumulated ^137^Cs more than males even after weight adjustment. Although we used virtual samples, this mathematical operation appeared to be reasonable based on *R*^2^ values and coefficients. The reasons for the sexual difference are not clear, but the difference in accumulation may stem from the difference in cellular metabolic rates: the cells in a female body may have generally higher metabolic rates than cells in a male body. Alternatively, but not exclusively, females may have higher cation concentrations in the hemolymph, and it may be hemolymph (but not muscle and other tissues) that accumulates ^137^Cs. These hypotheses may be tested by measuring metabolic rates or cation concentrations in hemolymph from both sexes. In any case, antioxidants for quenching ROS generated by irradiation may be depleted faster in females. This female-biased accumulation was true not only in the whole body but also in all body parts. The most accumulating organ in the body was the abdomen, occupying more than half of the entire radioactivity in the body. Female-biased accumulation was detected not only in the experimental ^137^Cs-fed butterflies but also in the field-collected butterflies, although the potential sex difference even in the September 2016 samples (35.7% versus 59.1%) was not statistically significant, not to mention the May 2011 and September 2011 samples. However, we detected significant difference between May 2011 and September 2016 only in females. In this sense, we speculate that the sexual differences are common in both the experimentally treated individuals and field individuals. Likely, females were more affected in Fukushima.

In a previous study, the next-generation butterflies from the first-generation females after the accident showed morphological abnormalities similar to those of the mother generation [16,17]. This adverse transgenerational (i.e., heritable) effect may be related to the high accumulation of not only ^137^Cs but also ^134^Cs, ^131^I, and other radionuclides in the reproductive organs, producing ROS. This speculation is reasonable if the accumulation process of various radionuclides is not very different among these radionuclides in butterflies, although ^110m^Ag and ^129m^Te have been reported to behave differently from ^137^Cs and ^134^Cs in cattle [55]. The adverse transgenerational effects may at least partly be considered maternal effects [86,87,88,89,90]. It seems that females may generally have higher sensitivity to environmental stress, including radiation. In fact, in the pale grass blue butterfly, females were reported to have higher morphological abnormality rates than males, even in noncontaminated areas [37]. Cold-shock-induced modifications are also female-biased in this butterfly species [32].

Accumulation in the abdomen, which was largest among the organs examined in this study, appears to be more notable at the posterior portion of the abdomen where reproductive organs are located, based on the PSL distribution images (Figure 1b). This may be because egg or sperm production requires high levels of cations, and cesium ions may be incorporated together with these cations. If so, high levels of ROS would be produced by β-rays from ^137^Cs and ^134^Cs, which may affect germ cells locally at the accumulated sites. The chemical behavior of ^131^I is unknown in butterflies, but it may have severe effects on organs. It should be noted that absorbed dose rate varies among radionuclides. Whether the amount of ROS produced by β-rays from ^137^Cs and ^134^Cs is large enough to affect germ cells and whether these cesium sources are more (or less) toxic than other nuclides including ^131^I are questions that should be answered in the future. Aside from the general fact that germ cells in meiosis are relatively error-prone to DNA damage and replication, and regardless of the relevance of this accumulation to genetic mutation, some individuals showed abnormal morphology in genitalia in an internal exposure experiment using the cabbage white butterfly *Pieris rapae* [20]. Upregulation or downregulation of ROS-related genes should be examined in the future.

Accumulation in the antennae and the head may affect the nervous system and, thus, animal behavior. In males, accumulation in the antennae may be notable, albeit *n* = 1 (Figure 6c). The highest accumulation was observed at the tip of an antenna (Figure 1b). If this accumulation causes neuronal damage, pheromone and odorant detection may be impaired. The antennae and legs are important sensory organs for determining the host plant. Additional experiments with more samples are required to solidify this point.

Accumulation in the thorax and legs may indicate accumulation in muscles. The thorax showed the second largest accumulation among those organs examined and the largest weight-adjusted sexual difference in the present study. Females likely have larger wing muscles in the thorax than males, possibly for a relatively long period of host plant search and oviposition behaviors. The results in this study are consistent with the accumulation of ^137^Cs in muscles in vertebrates [56,57,58,59,60,61,62,63,64,65] and earthworms [78].

Accumulation in wings may indicate accumulation in the cuticular surface (including scales, sockets, and basal membrane). Wing deposition of ^137^Cs may affect the expansion of wings immediately after eclosion; there were many individuals with abnormal wings, such as wrinkled wings, probably the most frequent morphological abnormality discovered after the accident. In the silkworm moth, pupal wings are sensitive to experimental irradiation [47,50].

### 4.3. No Condensation in Butterflies

Biological accumulation (condensation) of ^137^Cs through generations may be a potential source of biological effects, especially in short-lived animals such as the pale grass blue butterfly, although butterfly eggs may be too small to store much foreign substance. Such an accumulation was not detected in the present study; there was no significant difference between September 2011 samples and September 2016 samples at the time of measurements. To be sure, similar accumulation levels between September 2011 and September 2016 at the time of measurements made simultaneously in the present study indicate that the accumulation level of September 2011 at that time should be higher than that of September 2016 at that time because over five years, a portion of ^137^Cs in September 2011 must have decayed to become similar to the accumulation level in September 2016.

In addition to the experimental demonstration of transgenerational effects [16,17,40,41,42], a study using mathematical models (two dose reconstruction models based on the Gaussian plume model) concluded that these abnormalities are transgenerational effects caused by the initial exposure immediately after the accident [91]. However, this mathematical study could not exclude a possible effect from continuous radiation exposure in the contaminated environment. The present study concludes that a potential increase in internal exposure due to year-by-year accumulation in this butterfly is negligible. In addition, potential external exposure levels are decreasing in the environment. Considering these points, the models for transgenerational effects [91] are likely to be correct because a possible effect from continuous internal and external exposures can largely be excluded based on the present study.

Condensation of ^137^Cs in the body from a diet is also unlikely. According to our calculation, only 1.8% of the ingested ^137^Cs was assimilated into the body of a larva or pupa (Appendix E). Further one-tenth reduction (×0.1405) occurs upon eclosion to an adult butterfly, resulting in only 0.25% left in the adult bodies (Section 3.4 and Appendix E). We speculate that the biological half-life of ^137^Cs in this butterfly is probably just several days because most ^137^Cs would be consumed at the late fourth-instar stage, most of which would be excreted upon pupation and eclosion within several days. Excretion of potassium is likely an active process in butterflies [92,93], and thus, radioactive cesium ions may be excreted together with potassium ions. It seems that carnivorous arthropods accumulate higher levels of ^137^Cs through a food web [70,71,72,73,74,75,76,77], but such biological accumulation would not occur in herbivorous butterflies.

In the present study, we did not detect any individuals who had higher-than-control PSL values in the May 2011 samples. This result of the May 2011 samples is somewhat unexpected because it contrasts with the results of the September 2011 and September 2016 samples. However, the present results are consistent with abnormality dynamics, in which May 2011 shows a low abnormality rate, and September 2011 shows the highest rate in six surveys for three years [33]. We believe that this increase from May 2011 to September 2011 is not due to accumulation over generations. We speculate that the plant and overwintering larvae were probably under dried grass during the accident and even under accumulated snow in March 2011 in Fukushima [38]. We imagine that the direct and immediate absorption of radionuclides into plants was difficult under these conditions. In any case, the status of the first generation (May 2011) should be discussed elsewhere together with other available data.

## 5. Conclusions

The present study demonstrated the accumulation of anthropogenic ^137^Cs in the body of the pale grass blue butterfly. A portion of the experimentally ingested ^137^Cs at the larval stage was carried onto the adult body. Accumulation was female-biased, and the abdomen appeared to accumulate more ^137^Cs than other organs. Field-caught adult butterflies also showed accumulation, albeit at low levels. However, condensations of ^137^Cs in adult butterfly bodies over generations and from a contaminated diet were unlikely partly because the majority of ^137^Cs was excreted in the pupal cuticle and excretory material upon eclosion. Although this butterfly was shown to be resistant against the direct exposure effect of radiation [49], as in other insects, the accumulation in the abdomen suggests the possibility of an adverse ROS-mediated transgenerational (heritable) effect on germ cells, especially in females, even if direct DNA damage without DNA repair is probabilistically rare. This speculation is consistent with the adverse transgenerational effects detected in previous studies [16,17,39,40,41,42]. The present study will contribute to an integrative understanding of the biological impacts of the Fukushima nuclear accident.

## Figures and Tables

**Table 1:** Results of imaging plate autoradiography for field samples *^1^.

Sampling Month and Year	May 2011		September 2011		September 2016	
Sex	Male	Female	Male	Female	Male	Female
Number of individuals examined	6	7	8	11	28	22
Number of positive individuals with significantly larger PSL values *^2^	0	0	3	4	10	13
Percentage of positive individuals with significantly larger PSL values	0%	0%	37.5%	36.4%	35.7%	59.1%
Mean PSL ± SD *^3^	na	na	16,338 ± 6939	17,450 ± 2899	15,144 ± 3394	14,961 ± 4731
Maximum value of mean PSL	na	na	23,206 ± 8217	19,858 ± 9993	20,750 ± 8266	24,891 ± 5086

*^1^ Exposure period is 2 d in all samples listed in this table. *^2^ Significance: *p* < 0.05 (one-sided Welch’s *t*-test). *^3^ Mean PSL ± standard deviation. Only positive individuals with significantly larger PSL values were used to calculate the mean PSL. na: not applicable.

## Data Availability

All the data generated or analyzed during this study are included in this published article and its Supplementary Materials.

## Supplementary Materials

The following supporting information can be downloaded at https://www.mdpi.com/article/10.3390/life13051211/s1, as a single PDF file, which contains Table S1: Mean PSL values of the whole adult bodies, the pupal cuticle cases, and the whole pupal bodies; Table S2: Mean PSL values of the adult body parts; Table S3: Mean values of the adult weights. In addition, we have recently posted original pictures of field-collected butterflies with morphological abnormalities [94].

## Appendix A. Relationship between PSL and Becquerel

Here, we discuss the relationship between PSL and becquerel (Bq) because the former is dependent on the measurement system and because the latter is more familiar to readers. PSL is an arbitrary unit used to express the amount of luminescence induced by photons, including radiation. PSL values depend on exposure time, temperature, light intensity, and other conditions. Therefore, the relationship discussed below applies only to our system in this study.

According to Gurung et al. (2019) [49], a dried H6 prepupa had a ^137^Cs radioactivity concentration of 1.62 × 10^7^ Bq/kg (dry). In the present study, dried pupal weight was measured as 5.50 ± 1.30 mg (*n* = 10; mean ± standard deviation) using H0 individuals. Therefore, we obtained 89 Bq/pupa (or prepupa). In this study, exposure of an H6 pupa (dry) for 2 d (172,800 s) to an imaging plate resulted in 2.04 × 10^9^ PSL/pupa. Per second, it is 1.18 × 10^4^ PSL/pupa. This quantitative relationship between PSL and Bq was based on the following considerations.

First, the cuticular integument of larvae was shed when a prepupa metamorphosed into a pupa. This shed integument was not included in a pupal measurement. In the feeding experiment, the ^137^Cs-containing artificial diet was given mainly to the fourth-instar larvae, although the third-instar larvae may also be contained. Therefore, the prepupal integument of the fourth-instar larvae was made before the ingestion of ^137^Cs and thus does not contain ^137^Cs. Second, sexual distinction was not possible at the prepupal and pupal stages. As this study demonstrated, sex differences in terms of the accumulation of ^137^Cs are present, but random sampling would collect both males and females equally. Third, imaging plate analysis cannot distinguish radionuclides, but ^40^K can be considered equally distributed in all butterfly samples. Moreover, to obtain the relationship between PSL and becquerel above, H6 (the largest ^137^Cs radioactivity concentration among those tested in the previous study) was used, which made it possible to safely ignore radionuclides other than ^137^Cs; even if present, ^40^K was relatively negligible.

## Appendix B. Detection Limit of Imaging Plate Autoradiography Used in This Study

The sensitivity of imaging plate autoradiography is generally very high, and its practical “detection limit” is dependent on how reproducibly the system can discriminate “signal” from environmental or spontaneous “noise” (i.e., background signal). Here, we discuss how much radioactivity is required to discriminate signal from noise in the method used in this study.

We measured H0 adult individuals after 2 d exposure and obtained 10,701.18 ± 7167.55 PSL/adult (*n* = 3; mean ± standard deviation) in males and 14,460.54 ± 14,309.80 PSL/adult (*n* = 3; mean ± standard deviation) in females (Supplementary Table S1). These values were similar to and smaller than those of Table 1, confirming the feasibility of the analysis of field-caught individuals near the detection limit. Because both sexes had three individuals, we obtained 12,580.86 PSL/adult (*n* = 6) as the average of the two. This value was obtained after 2 d (172,800 s), and thus, an average adult individual had 0.07280590 PSL/s. This value represents a “background signal” from an average adult individual without minimal (supposedly almost no) exposure to ^137^Cs and other anthropogenic radionuclides.

On the other hand, based on Appendix A, we know that an H6 pupa had 11,800 PSL/s. At the same time, a pupa had 89 Bq. This means that 1.3 × 10^2^ PSL (=11,800/89) is obtained per Bq. Thus, the “background signal” obtained above, 0.07280590 PSL/s, can be converted to 5.5 × 10^−4^ Bq (=0.07280590 × (89/11,899)), which is considered the detection limit of the current method.

## Appendix C. Relationship between the Whole Body and Body Parts

Theoretically, summation of the PSL values from all the body parts should be equal to the PSL value of the original whole body. We tested this by obtaining the PSL ratio (whole body divided by summation of body parts). The ratio was 1.2 ± 0.3 (*n* = 4), justifying minimum loss of PSL signals associated with dissection.

## Appendix D. Feasibility for Detecting ^137^Cs in Field Samples

Because the PLS values of the field samples were at the verge of the detection limit of the system, the feasibility of our methods and the reliability of the output values should be discussed. Here, we compare the positive individuals of the field samples (September 2011) with the H3 samples. These two groups were internally subjected to radiation exposure in the field (the September 2011 samples) or in the laboratory (the H3 samples) roughly at comparative levels [49]. Furthermore, to obtain PSL values, both were exposed on an imaging plate for 2 d. The mean PSL values of the September 2011 samples were 16,338 PSL (*n* = 3) in males and 17,450 PSL (*n* = 4) in females (Table 1), whereas those of the experimental H3 samples were 40,284 PSL (*n* = 3) in males and 126,582 PSL (*n* = 3) in females (Supplementary Table S1).

The radioactivity concentration of ^137^Cs in wild host plant leaves collected at Hanamiyama, Fukushima City, in July 2011 was reported to be 4210 Bq/kg (wet) [39]. We may consider that leaves with similar levels of contamination were consumed by larvae in the field in September 2011. On the other hand, the concentration in the H3 artificial diet is rigorously known as 45,500 Bq/kg (wet) [49]. That is, the accumulation of radioactivity in the September 2011 field individuals should have approximately one-tenth (4210/45,500 = 0.09253) of the experimental H3 if the assimilation process of ^137^Cs to the body is not different between the wild leaves and the artificial diet.

Along this line of argument, we can estimate the expected mean PSL values of the field samples from those of the H3 samples, assuming linearity. The mean PLS values of September 2011 samples should theoretically be 3727 PSL (= 40,284 × 0.09253) in males and 11,713 PSL (=126,582 × 0.09253) in females. These theoretical values are smaller than the experimental values detected, but in females, the detected and estimated values are on the same order of magnitude. The discrepancy in males may simply be due to small sample size but is not well understood. Overall, we conclude that the PLS values of the field samples were not rigorous but still contained useful information regarding ^137^Cs accumulation in the body.

## Appendix E. Assimilation Rate and Adult Retention Rate

Using the relationship in Appendix A, it is possible to estimate the “assimilation rate” of ^137^Cs from the ingested ^137^Cs-containing artificial diet to the body. Assuming that the prepupal (larval) cuticle is negligible in weight and that the prepupal (larval) and pupal weights are identical, 89 Bq/pupa is the radioactivity concentration of an H6 pupa. We know that an H6 larva consumed 4940 Bq/larva [49]. Therefore, the assimilation rate from the consumed diet to the pupal or larval body was 1.8% (=(89/4940) × 100).

To calculate how much radioactivity is retained in the adult bodies after excretion in the pupal cuticle and excretory material, we first obtained PSL values of the adult whole bodies at each ingestion level (H3–H6). Because PSL values of the adult whole bodies were known in two sexes independently, we used mean values of two sexes as a representative PSL value for a given ingestion level. At each ingestion level, this value of the adult whole bodies was divided by that of the pupa. The mean and SD values of these adult/pupa ratios from H3 to H6 were calculated to be 0.1405 ± 0.0294 (*n* = 4), which was considered the adult retention rate. Therefore, the adult bodies retained 0.25% (=1.8% × 0.1405) of the radioactivity in the consumed diet (Section 3.4).

## References

[1] Chino M., Nakayama H., Nagai H., Terada H., Katata G., Yamazawa H. (2011). Preliminary estimation of released amount of ^131^I and ^137^Cs accidentally discharged from the Fukushima Daiichi nuclear power plant into the atmosphere. J. Nucl. Sci. Technol..

[2] Kinoshita N., Sucki K., Sasa K., Kitagawa J., Ikarashi S., Nishimura T., Wong Y.S., Satou Y., Handa K., Takahashi T. (2011). Assessment of individual radionuclide distributions from the Fukushima nuclear accident covering central-east Japan. Proc. Natl. Acad. Sci. USA.

[3] Hirose K. (2012). 2011 Fukushima Dai-ichi nuclear power plant accident: Summary of regional radioactive deposition monitoring results. J. Environ. Radioact..

[4] Torii T., Sugita T., Okada C.E., Reed M.S., Blumenthal D.J. (2013). Enhanced analysis methods to derive the spatial distribution of ^131^I deposition on the ground by airborne surveys at an early stage after the Fukushima Daiichi nuclear power plant accident. Health Phys..

[5] Le Petit G., Douysset G., Ducros G., Gross P., Achim P., Monfort M., Raymond P., Pontillon Y., Jutier C., Blanchard X. (2014). Analysis of radionuclide releases from the Fukushima Dai-ichi nuclear power plant accident Part I. Pure Appl. Geophys..

[6] Achim P., Monfort M., Le Pettit G., Gross P., Douysset G., Taffary T., Blanchard X., Moulin C. (2014). Analysis of radionuclide releases from the Fukushima Dai-ichi nuclear power plant accident Part II. Pure Appl. Geophys..

[7] Ministry of the Environment Chapter 4. Radiation Protection. 4.4 Long-Term Effects. Posted on 31 March 2016. English Version. https://www.env.go.jp/en/chemi/rhm/basic-info/1st/04-04-08.html.

[8] Lokobauer N., Franić Z., Bauman A., Maračić M., Cesar D., Senčar J. (1998). Radiation contamination after the Chernobyl nuclear accident and the effective dose received by the population of Croatia. J. Environ. Radioact..

[9] Arapis G.D., Karandinos M.G. (2004). Migration of ^137^Cs in the soil of sloping semi-natural ecosystems in Northern Greece. J. Environ. Radioact..

[10] Tahir S.N.A., Jamil K., Zaidi J.H., Arif M., Ahmed N. (2006). Activity concentration of ^137^Cs in soil samples from Punjab province (Pakistan) and estimation of gamma-ray dose rate for external exposure. Radiat. Prot. Dosim..

[11] Ambrosino F., Stellato L., Sabbarese C. (2020). A case study on possible radiological contamination in the Lo Uttara landfill site (Caserta, Italy). J. Phys. Conf. Ser..

[12] Endo S., Kimura S., Takatsuji T., Nanasawa K., Imanaka T., Shizuma K. (2021). Measurement of soil contamination by radionuclides due to the Fukushima Dai-ichi Nuclear Ppower Plant accident and associated estimated cumulative external dose estimation. J. Environ. Radioact..

[13] Møller A.P., Hagiwara A., Matsui S., Kasahara S., Kawatsu K., Nishiumi I., Suzuki H., Mousseau T.A. (2012). Abundance of birds in Fukushima as judges from Chernobyl. Environ. Pollut..

[14] Murase K., Murase J., Horie R., Endo K. (2015). Effects of the Fukushima Daiichi nuclear accident on goshawk reproduction. Sci. Rep..

[15] Bonisoli-Alquati A., Koyama K., Tedeschi D.J., Kitamura W., Sukuzi H., Ostermiller S., Arai E., Møller A.P., Mousseau T.A. (2015). Abundance and genetic damage of barn swallows from Fukushima. Sci. Rep..

[16] Hiyama A., Nohara C., Kinjo S., Taira W., Gima S., Tanahara A., Otaki J.M. (2012). The biological impacts of the Fukushima nuclear accident on the pale grass blue butterfly. Sci. Rep..

[17] Hiyama A., Nohara C., Taira W., Kinjo S., Iwata M., Otaki J.M. (2013). The Fukushima nuclear accident and the pale grass blue butterfly: Evaluating biological effects of long-term low-dose exposures. BMC Evol. Biol..

[18] Akimoto S. (2014). Morphological abnormalities in gall-forming aphids in a radiation-contaminated area near Fukushima Daiichi: Selective impact of fallout?. Ecol. Evol..

[19] Akimoto S.I., Li Y., Imanaka T., Sato H., Ishida K. (2018). Effects of radiation from contaminated soil and moss in Fukushima on embryogenesis and egg hatching of the aphid *Prociphilus oriens*. J. Hered..

[20] Taira W., Toki M., Kakinohana K., Sakauchi K., Otaki J.M. (2019). Developmental and hemocytological effects of ingesting Fukushima’s radiocesium on the cabbage white butterfly *Pieris rapae*. Sci. Rep..

[21] Ochiai K., Hayama S., Nakiri S., Nakanishi S., Ishii N., Uno T., Kato T., Konno F., Kawamoto Y., Tsuchida S. (2014). Low blood cell counts in wild Japanese monkeys after the Fukushima Daiichi nuclear disaster. Sci. Rep..

[22] Hayama S., Tsuchiya M., Ochiaki K., Nakiri S., Nakanishi S., Ishii N., Kato T., Tanaka A., Konno F., Kawamoto Y. (2017). Small head size and delayed body weight growth in wild Japanese monkey fetuses after the Fukushima Daiichi nuclear disaster. Sci. Rep..

[23] Urushihara Y., Suzuki T., Shimizu Y., Ohtaki M., Kuwahara Y., Suzuki M., Uno T., Fujita S., Saito A., Yamashiro H. (2018). Haematological analysis of Japanese macaques (*Macaca fuscata*) in the area affected by the Fukushima Daiichi Nuclear Power Plant accident. Sci. Rep..

[24] Horiguchi T., Yoshii H., Mizuno S., Shiraishi H. (2016). Decline in intertidal biota after the 2011 Great East Japan Earthquake and Tsunami and the Fukushima nuclear disaster: Field observations. Sci. Rep..

[25] Hayashi G., Shibato J., Imanaka T., Cho K., Kubo A., Kikuchi S., Satoh K., Kimura S., Ozawa S., Fukutani S. (2014). Unraveling low-level gamma radiation-responsive changes in expression of early and late genes in leaves of rice seedlings at Iitate Village, Fukushima. J. Hered..

[26] Watanabe Y., Ichikawa S., Kubota M., Hoshino J., Kubota Y., Maruyama K., Fuma S., Kawaguchi I., Yoschenko V.I., Yoshida S. (2015). Morphological defects in native Japanese fir trees around the Fukushima Daiichi Nuclear Power Plant. Sci. Rep..

[27] Yoschenko V., Nanba K., Yoshida S., Watanabe Y., Takase T., Sato N., Keitoku K. (2016). Morphological abnormalities in Japanese red pine (*Pinus densiflora*) at the territories contaminated as a result of the accident at Fukushima Dai-ichi Nuclear Power Plant. J. Environ. Radioact..

[28] Shirôzu T. (2006). The Standard of Butterflies in Japan.

[29] Yata O. (2007). Iconographia Insectorum Japonicorum Colore Naturali Esita Vol. I.

[30] Oda H., Kitazoe N. (2002). Observation Encyclopedia of Lycaenid Butterflies.

[31] Hiyama A., Iwata M., Otaki J.M. (2010). Rearing the pale grass blue *Zizeeria maha* (Lepidoptera, Lycaenidae): Toward the establishment of a lycaenid model system for butterfly physiology and genetics. Entomol. Sci..

[32] Otaki J.M., Hiyama A., Iwata M., Kudo T. (2010). Phenotypic plasticity in the range-margin population of the lycaenid butterfly *Zizeeria maha*. BMC Evol. Biol..

[33] Hiyama A., Taira W., Nohara C., Iwasaki M., Kinjo S., Iwata M., Otaki J.M. (2015). Spatiotemporal abnormality dynamics of the pale grass blue butterfly: Three years of monitoring (2011–2013) after the Fukushima nuclear accident. BMC Evol. Biol..

[34] Sakauchi K., Taira W., Hiyama A., Imanaka T., Otaki J.M. (2020). The pale grass blue butterfly in ex-evacuation zones 5.5 years after the Fukushima nuclear accident: Contributions of initial high-dose exposure to transgenerational effects. J. Asia-Pac. Entomol..

[35] Taira W., Iwasaki M., Otaki J.M. (2015). Body size distributions of the pale grass blue butterfly in Japan: Size rules and the status of the Fukushima population. Sci. Rep..

[36] Hiyama A., Taira W., Iwasaki M., Sakauchi K., Gurung R., Otaki J.M. (2017). Geographical distribution of morphological abnormalities and wing color pattern modifications of the pale grass blue butterfly in northeastern Japan. Entomol. Sci..

[37] Hiyama A., Taira W., Iwasaki M., Sakauchi K., Iwata M., Otaki J.M. (2017). Morphological abnormality rate of the pale grass blue butterfly *Zizeeria maha* (Lepidoptera: Lycaenidae) in southwestern Japan: A reference data set for environmental monitoring. J. Asia-Pac. Entomol..

[38] Sakauchi K., Taira W., Toki M., Iraha Y., Otaki J.M. (2019). Overwintering states of the pale grass blue butterfly *Zizeeria maha* (Lepidoptera: Lycaenidae) at the time of the Fukushima nuclear accident in March 2011. Insects.

[39] Nohara C., Hiyama A., Taira W., Tanahara A., Otaki J.M. (2014). The biological impacts of ingested radioactive materials on the pale grass blue butterfly. Sci. Rep..

[40] Nohara C., Taira W., Hiyama A., Tanahara A., Takatsuji T., Otaki J.M. (2014). Ingestion of radioactively contaminated diets for two generations in the pale grass blue butterfly. BMC Evol. Biol..

[41] Taira W., Hiyama A., Nohara C., Sakauchi K., Otaki J.M. (2015). Ingestional and transgenerational effects of the Fukushima nuclear accident on the pale grass blue butterfly. J. Radiat. Res..

[42] Nohara C., Hiyama A., Taira W., Otaki J.M. (2018). Robustness and radiation resistance of the pale grass blue butterfly from radioactively contaminated areas: A possible case of adaptive evolution. J. Hered..

[43] Otaki J.M. (2016). Fukushima’s lessons from the blue butterfly: A risk assessment of the human living environment in the post-Fukushima era. Integr. Environ. Assess. Manag..

[44] Otaki J.M., Taira W. (2018). Current status of the blue butterfly in Fukushima research. J. Hered..

[45] Otaki J.M., Fukumoto M. (2020). The pale grass blue butterfly as an indicator for the biological effect of the Fukushima Daiichi Nuclear Power Plant accident. Low-Dose Radiation Effects on Animals and Ecosystems.

[46] Otaki J.M., Sakauchi K., Taira W. (2022). The second decade of the blue butterfly in Fukushima: Untangling the ecological field effects after the Fukushima nuclear accident. Integr. Environ. Assess. Manag..

[47] Takada N., Yamauchi E., Fujimoto H., Banno Y., Tsuchida K., Hashido K., Nakajima Y., Tu Z., Takahashi M., Fujii H. (2006). A novel indicator for radiation sensitivity using the wing size reduction of Bombyx mori pupae caused by γ-ray irradiation. J. Insect Biotechnol. Sericol..

[48] Ahmad S., Hussain A., Ullah F., Jamil M., Ali A., Ali S., Luo Y. (2021). ^60^Co-γ radiation alters developmental stages of *Zeugodacus cucurbitae* (Diptera: Tephritidae) through apoptosis pathways gene expression. J. Insect Sci..

[49] Gurung R.D., Taira W., Sakauchi K., Iwata M., Hiyama A., Otaki J.M. (2019). Tolerance of high oral doses of nonradioactive and radioactive caesium chloride in the pale grass blue butterfly *Zizeeria maha*. Insects.

[50] Tanaka S., Kinouchi T., Fujii T., Imanaka T., Takahashi T., Fukutani S., Maki D., Nohtomi A., Takahashi S. (2020). Observation of morphological abnormalities in silkworm pupae after feeding ^137^CsCl-supplemented diet to evaluate the effects of low dose-rate exposure. Sci. Rep..

[51] Otaki J.M., Awwad N.S., AlFaify S.A. (2018). Understanding low-dose exposure and field effects to resolve the field-laboratory paradox: Multifaceted biological effects from the Fukushima nuclear accident. New Trends in Nuclear Science.

[52] Sakauchi K., Taira W., Toki M., Tsuhako M., Umetsu K., Otaki J.M. (2021). Nutrient imbalance of the host plant for larvae of the pale grass blue butterfly may mediate the field effect of low-dose radiation exposure in Fukushima: Dose-dependent changes in the sodium content. Insects.

[53] Sakauchi K., Taira W., Otaki J.M. (2021). Metabolomic response of the creeping wood sorel *Oxalis corniculata* to low-dose radiation exposure from Fukushima’s contaminated soil. Life.

[54] Sakauchi K., Taira W., Otaki J.M. (2022). Metabolomic profiles of the creeping wood sorel *Oxalis corniculata* in radioactively contaminated fields in Fukushima: Dose-dependent changes in key metabolites. Life.

[55] Morita A., Sakauchi K., Taira W., Otaki J.M. (2022). Ingestional toxicity of radiation-dependent metabolites of the host plant for the pale grass blue butterfly: A mechanism of field effects of radioactive pollution in Fukushima. Life.

[56] Fukuda T., Kino Y., Abe Y., Yamashiro H., Kuwahara Y., Nihei H., Sano Y., Irisawa A., Shimura T., Obata Y. (2013). Distribution of artificial radionuclides in abandoned cattle in the evacuation zone of the Fukushima Daiichi nuclear power plant. PLoS ONE.

[57] Sato I., Okada K., Sasaki J., Chida H., Satoh H., Miura K., Kikuchi K., Otani K., Sato S. (2015). Distribution of radioactive cesium and stable cesium in cattle kept on a highly contaminated area of Fukushima nuclear accident. Anim. Sci. J..

[58] Fuma S., Kubota Y., Ihara S., Takahashi H., Watanabe Y., Aono T., Soeda H., Yoshida S. (2016). Radiocaesium contamination of wild boars in Fukushima and surrounding regions after the Fukushima nuclear accident. J. Environ. Radioact..

[59] Tanoi K., Uchida K., Doi C., Nihei N., Hirose A., Kobayashi N.I., Sugita R., Nobori T., Nakanishi T.M., Kanno M. (2016). Investigation of radiocesium distribution in organs of wild boar grown in Iitate, Fukushima after the Fukushima Daiichi nuclear power plant accident. J. Radioanal. Nucl. Chem..

[60] Saito R., Nemoto Y., Tsukada H. (2020). Relationship between radiocaesium in muscle and physicochemical fractions of radiocaesium in the stomach of wild boar. Sci. Rep..

[61] Morimoto M., Kobayashi J., Kino Y. (2022). Radiation dose and gene expression analysis of wild boar 10 years after the Fukushima Daiichi Nuclear Plant accident. Sci. Rep..

[62] Hayama S., Nakiri S., Nakanishi S., Ishii N., Uno T., Kato T., Konno F., Kawamoto Y., Tsuchida S., Ochiai K. (2013). Concentration of radiocesium in the wild Japanese manokey (*Macaca fuscata*) over the first 15 months after the Fukushima Daiichi nuclear disaster. PLoS ONE.

[63] Omi T., Nakiri S., Nakanishi S., Ishii N., Uno T., Konno F., Inagaki T., Sakamoto A., Shito M., Udagawa C. (2020). Concentrations of ^137^Cs radiocaesium in the organs and tissues of low-dose-exposed wild Japanese monkeys. BMC Res. Notes.

[64] Tsuboi J., Abe S., Fujimoto K., Kaeriyama H., Ambe D., Matsuda K., Enomoto M., Tomiya A., Morita T., Ono T. (2015). Exposure of a herbivorous fish to ^134^Cs and ^137^Cs from the riverbed following the Fukushima disaster. J. Environ. Radioact..

[65] Ishii N., Furota T., Kagami M., Tagami K., Uchida S. (2021). Inequality in the distribution of ^137^Cs contamination within freshwater fish bodies and its affecting factors. Sci. Rep..

[66] Yoshimura M., Akama A. (2014). Radioactive contamination of aquatic insects in a stream impacted by the Fukushima nuclear power station accident. Hydrobiologia.

[67] Ishii Y., Miura H., Jo J., Tsuji H., Saito R., Koarai K., Hagiwara H., Urushidate T., Nishikiori T., Wada T. (2022). Radiocesium-bearing microparticles cause a large variation in ^137^Cs activity concentration in the aquatic insect *Stenopsyche marmorata* (Tricoptera: Stenopsychidae) in the Ota River, Fukushima, Japan. PLoS ONE.

[68] Iwasa M., Nakaya F., Kabeya H., Sato K., Ishikawa S.-I., Takahashi T. (2020). Radiocesium concentrations in invertebrates and their environmental media at two distances from the Fukushima Dai-ichi Nuclear Power Plant during 3-6 years after the 2011 accident. Environ. Pollut..

[69] Fuma S., Ihara S., Takahashi H., Inaba O., Sato Y., Kubota Y., Watanabe Y., Kawaguchi I., Aono T., Soeda H. (2017). Radiocaesium contamination and dose rate estimation of terrestrial and freshwater wildlife in the exclusion zone of the Fukushima Dai-ichi Nuclear Power Plant accident. J. Environ. Radioact..

[70] Ayabe Y., Kanasashi T., Hijii N., Takenaka C. (2014). Radiocesium contamination of the web spider *Nephila clavate* (Nephilidae: Arachnida) 1.5 years after the Fukushima Dai-ichi Nuclear Power Plant accident. J. Envion. Radioact..

[71] Sasaki Y., Ishii Y., Abe H., Mitachi K., Watanabe T., Niizato T. (2017). Translocation of radiocesium released by the Fukushima Daiichi Nuclear Power Plant accident in Japanese chestnut and chestnut weevil larvae. Hortic. J..

[72] Murakami M., Ohte N., Suzuki T., Ishii N., Igarashi Y., Tanoi K. (2014). Biological proliferation of cesium-137 through the detrital food chain in a forest ecosystem in Japan. Sci. Rep..

[73] Tanaka S., Hatakeyama K., Takahashi S., Adati T. (2016). Radioactive contamination of arthropods from different trophic levels in hilly and mountainous areas after the Fukushima Daiichi nuclear power plant accident. J. Environ. Radioact..

[74] Ishii Y., Hayashi S., Takamura N. (2017). Radiocesium transfer in forest insect communities after the Fukushima Dai-ichi Nuclear Power Plant accident. PLoS ONE.

[75] Iwasa M., Sato K., Ishikawa S., Takahashi T., Kabeya H., Nakaya F. (2022). Radiocesium contaminations and transfer in cyclorrhaphous flies (Diptera: Muscidae, Calliphoridae) at three distances from the Fukushima Dai-ichi Nuclear Power Plant after the 2011 accident. Appl. Entomol. Zool..

[76] Nakanishi H., Mori A., Takeda K., Tanaka H., Kobayashi N., Tanoi K., Yamakawa T., Mori S. (2015). Discovery of radioactive silver (^110m^Ag) in spiders and other fauna in the terrestrial environment after the meltdown of Fukushima Dai-ichi nuclear power plant. Proc. Jpn. Acad. Ser. B.

[77] Ayabe Y., Yoshida T., Kanasashi T., Hayashi A., Fukushi A., Hijii N., Takenaka C. (2019). Web-building spider *Nephila clavate* (Nephilidae: Arachnida) can represent ^137^Cs contamination of arthropod communities and bioavailable ^137^Cs in forest soil at Fukushima, Japan. Sci. Total Environ..

[78] Tanaka S., Adati T., Takahashi T., Fujiwara K., Takahashi S. (2018). Concentrations and biological half-life of radioactive cesium in epigeic earthworms after the Fukushima Dai-ichi Nuclear Power Plant accident. J. Environ. Radioact..

[79] Murakami H., Hatta T., Kitazawa H., Yamada H., Yaita T., Kogure T. (2014). Speciation of radioactive soil particles in the Fukushima contaminated area by IP autoradiography and microanalyses. Environ. Sci. Technol..

[80] Nakanishi T.M., Kobayashi N.I., Tanoi K. (2013). Radioactive cesium deposition on rice, wheat, peach tree and soil after nuclear accident in Fukushima. J. Radioanal. Nucl. Chem..

[81] Nakanishi T.M. (2016). Agricultural implications of the Fukushima nuclear accident. J. Radiat. Res..

[82] Furuta E. (2013). Semi-quantitative analysis of leaf surface contamination by radioactivity from the Fukushima Daiichi nuclear power plant accident using HPGe and imaging plate. J. Radioanal. Nucl. Chem..

[83] Miura T., Mimura M., Kobayashi D., Komiyama C., Sekimoto H., Miyamoto M., Kitamura A. (2014). Radioactive pollution and accumulation of radionuclides in wild plants in Fukushima. J. Plant. Res..

[84] Ikka T., Nishina Y., Kamoshita M., Oya Y., Okuno K., Morita A. (2018). Radiocesium uptake through leaf surfaces of tea plants (*Camellia sinensis* L.). J. Environ. Radioact..

[85] Yamaguchi T., Sawano K., Furuhama K., Mori C., Yamada K. (2013). An autoradiogram of skeletal muscle from a pig raised on a farm within 20 km of the Fukushima Daiichi Nuclear Power Plant. J. Vet. Med. Sci..

[86] Mousseau T.A., Dingle H. (1991). Maternal effects in insect life histories. Annu. Rev. Entomol..

[87] Mousseau T., Fox C.W. (1998). The adaptive significance of maternal effects. Trends Ecol. Evol..

[88] Uller T. (2008). Developmental plasticity and the evolution of parental effects. Trends Ecol. Evol..

[89] Woestmann L., Saastamoinen M. (2016). The importance of trans-generational effects in Lepidoptera. Curr. Zool..

[90] Yoshida A., Yabu S., Otaki J.M. (2023). The plastic larval body color of the pale grass blue butterfly *Zizeeria maha* (Lepidoptera: Lycaenidae) in response to the host plant color: The maternal effect on crypsis. Insects.

[91] Hancock S., Vo N.T.K., Omar-Nazir L., Batlle J.V.I., Otaki J.M., Hiyama A., Byun S.H., Seymour C.B., Mothersill C. (2019). Transgenerational effects of historic radiation dose in pale grass blue butterflies around Fukushima following the Fukushima Dai-ichi Nuclear Power Plant meltdown accident. Environ. Res..

[92] Strathie L.W., Nicolson S.W. (1993). Post-eclosion diuresis in a flightless insect, the silkmoth *Bombyx mori*. Physiol. Entomol..

[93] Inoue T.A., Ito T., Hagiya H., Hata T., Asaoka K., Yokohari F., Niihara K. (2015). K^+^ excretion: The other purpose for puddling behavior in Japanese *Papilio* butterflies. PLoS ONE.

[94] Sakauchi K., Taira W., Otaki J.M. (2023). Instruction, Table, Picture Sheet, and Original Pictures: Morphological Abnormalities in the Field Samples of the Pale Grass Blue Butterfly for Three Years after the Fukushima Nuclear Accident. https://figshare.com/collections/Instruction_table_picture_sheet_and_original_pictures_Morphological_abnormalities_in_the_field_samples_of_the_pale_grass_blue_butterfly_for_three_years_after_the_Fukushima_nuclear_accident/6425981.

