# Supplementary material for: Imaging Plate Autoradiography for Ingested Anthropogenic Cesium-137 in Butterfly Bodies: Implications for the Biological Impacts of the Fukushima Nuclear Accident
Source: Life (Basel). 2023 May 18;13(5):1211. doi: 10.3390/life13051211 (PMC10222702; doi:10.3390/life13051211)
Supplement: Supplementary file 1 [file life-13-01211-s001.zip › life-2344660-supplementary.pdf]

Supplementary Materials

Imaging Plate Autoradiography for Ingested Anthropogenic Cesium-137 in Butterfly Bodies: Implications for the Biological Impacts of the Fukushima Nuclear Accident

Ko Sakauchi and Joji M. Otaki *

The BCPH Unit of Molecular Physiology, Department of Chemistry, Biology and Marine Science, Faculty of Sci-ence, University of the Ryukyus, Nishihara, Okinawa 903-0213, Japan

* Correspondence: otaki@sci.u-ryukyu.ac.jp; Tel.: +81-98-895-8557

Supplementary Tables S1, S2, and S3

Supplementary Table S1 Mean PSL values of the whole adult bodies, the pupal cuticle cases, and the whole pupal bodies

			Mean PSL ± SD (*n*)				
		Sex	One day	Two days	Four days	Six days	Eight days
Adult body	H0	M	7,029 ± 2,776 (3)	10,701 ± 7,168 (3)	65,039 ± 29,931 (3)	65,385 ± 33,475 (3)	119,647 ± 71,876 (3)
		F	10,746 ± 5,705 (3)	14,461 ± 14,310 (3)	95,270 ± 17,267 (3)	182,120 ± 31,953 (3)	191,163 ± 37,786 (3)
	H3	M	19,165 ± 7,108 (3)	40,284 ± 12,229 (3)	158,591 ± 43,752 (3)	263,431 ± 79,816 (3)	366,442 ± 137,552 (3)
		F	39,642 ± 21,307 (3)	126,582 ± 32,352 (3)	460,280 ± 184,681 (3)	810,413 ± 259,789 (3)	1,173,289 ± 439,976 (3)
	H4	M	195,493 ± 58,919 (3)	465,275 ± 134,602 (3)	1,428,086 ± 451,412 (3)	2,483,090 ± 768,203 (3)	4,124,153 ± 1,308,203 (3)
		F	534,350 ± 107,045 (3)	1,472,489 ± 252,757 (3)	3,765,216 ± 767,551 (3)	6,998,388 ± 1,604,039 (3)	11,524,425 ± 2,935,287 (3)
	H5	M	1,018,049 ± 1,002,956 (3)	2,148,531 ± 1,553,521 (3)	6,763,006 ± 5,645,805 (3)	6,189,087 ± 9,642,053 (3)	18,333,064 ± 15,106,715 (3)
		F	13,093,216 ± 10,066,089 (3)	30,604,654 ± 22,931,175 (3)	70,919,911 ± 51,879,874 (3)	114,649,272 ± 82,598,135 (3)	161,330,743 ± 113,471,727 (3)
	H6	M	46,899,765 ± 73,540,732 (3)	96,710,009 ± 147,211,478 (3)	208,441,315 ± 309,763,047 (3)	327,712,480 ± 478,447,671 (3)	483,780,603 ± 698,145,398 (3)
		F	232,609,445 ± 23,017,823 (3)	498,706,390 ± 62,463,030 (3)	1,024,319,754 ± 117,281,705 (3)	1,572,196,188 ± 155,569,884 (3)	2,116,998,550 ± 158,964,768*[1] (3)
Pupal case*[2]	H0	M	—	11,993 ± 9,053 (3)	—	—	—
		F	—	25,558 ± 5,091 (3)	—	—	—
	H3	M	—	65,954 (1)	—	—	—
		F	—	178,673 ± 22,733 (3)	—	—	—
	H4	M	—	738,088 ± 563,246 (3)	—	—	—
		F	—	1,274,027 ± 423,189 (3)	—	—	—
	H5	M	—	17,661,038 (1)	—	—	—
		F	—	48,214,774 ± 31,486,430 (3)	—	—	—
	H6	M	—	—	—	—	—
		F	—	450,357,498 ± 119,145,968 (3)	—	—	—

Pupa	H0	UK*[3]	—	123,516 ± 20,135 (3)	—	—	—
	H3	UK	—	812,615 ± 194,991 (3)	—	—	—
	H4	UK	—	6,693,791 ± 1,871,682 (3)	—	—	—
	H5	UK	—	97,274,367 ± 24,257,439 (3)	—	—	—
	H6	UK	—	2,038,394,060 ± 883,388,380 (3)	—	—	—

Numbers of individuals examined are indicated in parentheses. Mean ± standard deviation values are shown.

*[1]: Females of H6 for the eight-day exposure were beyond the maximum detection limit.

*[2]: The pupal cases were obtained after the eclosion to the adults but not after the pupation.

*[3]: Sex of pupae is unknown.

—: Not applicable.

Supplementary Table S2 Mean PSL values of the adult body parts

	Sex	Mean PSL for 2-d exposure ± SD (*n*)					
		Wings*[1]	Antennae	Legs*[2]	Head	Thorax	Abdomen
H6	M	1,372,869 ± 591,363 (3)	1,445,011 (1)	703,834 ± 765,335 (2)	1,121,807 ± 602,136 (3)	2,582,828 ± 2,562,044 (3)	78,380,317 ± 127,653,010 (3)
	F	5,268,543 ± 5,068,217 (3)	3,114,719 ± 1,170,374 (3)	2,970,962 ± 213,062 (2)	7,135,176 ± 4,691,791 (3)	30,487,767 ± 19,243,246 (3)	320,493,204 ± 93,573,185 (3)

Numbers of individuals examined are indicated in parentheses. Mean ± standard deviation values are shown.
*[1]: Wings indicate the sum of the four wings.
*[2]: Legs indicate the sum of the six legs.

Supplementary Table S3 Mean values of the adult weights

		Mean mg ± SD (*n*)							
	Sex	Whole body	Pupal case	Wings*[2]	Antennae	Legs*[3]	Head	Thorax	Abdomen
H0*[1]	M	3.91 ± 0.94 (7)	0.84 ± 0.85 (7)	1.03 ± 0.17 (8)	0.08 ± 0.02 (7)	0.15 ± 0.03 (5)	0.24 ± 0.03 (8)	1.21 ± 0.27 (5)	1.06 ± 0.45 (5)
	F	6.58 ± 0.84 (7)	0.86 ± 1.02 (7)	1.27 ± 0.15 (8)	0.12 ± 0.01 (7)	0.22 ± 0.02 (6)	0.24 ± 0.02 (7)	1.78 ± 0.24 (5)	2.56 ± 0.66 (5)

Numbers of individuals examined are indicated in parentheses. Mean ± standard deviation values are shown.

*[1]: These samples were used only for measuring weights and not for IP exposures.

*[2]: Wings indicate the sum of the four wings.

*[3]: Legs indicate the sum of the six legs.
